# Supplementary material for: Patterns of Intron Gain and Loss in Fungi
Source: PLoS Biol. 2004 Nov 30;2(12):e422. doi: 10.1371/journal.pbio.0020422 (PMC532390; doi:10.1371/journal.pbio.0020422)
Supplement: Table S1 — Also available at http://genes.mit.edu/NielsenEtAl/. (4.3 MB ZIP). [file pbio.0020422.st001.zip › NielsenEtAl/html/867.html]

AN1733.1.NCU03076.1.MG00189.1.FG01141.1


```
 CLUSTAL W (1.82) Multiple Sequence Alignments - Introns Inserted


Sequence 1: NCU03076.1	601 aa
Sequence 2: FG01141.1	576 aa
Sequence 3: MG00189.1	600 aa
Sequence 4: AN1733.1	572 aa
Alignment Length: 607 aa
Number Identitical Residues: 304 aa
Alignment Score (without introns) 14624


MG00189.1 	MALTRLGLTLTRSSRLALRPSYSSPLPQQLRRAPSGLR------QISTSQIRMSTIASFK
NCU03076.1	MSARRL-QRLSLTTSSGLRSARTSSTTINSALLRPQLYNQRSRTAIPSIVRTNSTLATFK
FG01141.1 	MSS------MMFLNRAGARGLRTAARTQQ--------------------MRTAATLATFK
AN1733.1  	---------MQSSMLLRVRALPKTASVLS--------------------RTKTATYATYK
          	         :        *   .:.   .                        :* *::*

MG00189.1 	IPKVVNEPN0QHYEKGSPSREGLTAALSALKQKGAVEVPIVVGGKE0IKTSSTGTQVNPA
NCU03076.1	VPTVANEPN0QHYAKGSESRHKLTEAYTALSKNLPVDVPINIRGRT0LSKADTKYQVNPA
FG01141.1 	TPKVYNEPN~QHYTKGSEQRQGLTAAIEKLQKQLPIEVPVVVGGKE0IKASALSKQQNPA
AN1733.1  	VPRIDNEPN0KHYAAGSPDRKALQEALARTQRNAPLSVPLVIAGKE0VKSSSSLTQSNPA
          	 * : **** :**  ** .*. *  *    .:: .:.**: : *:  :. :    * ***

MG00189.1 	DHQTVIATYSKASPEDVSRAIDEALAAKKDWESLPFNDRAAIFLKAADLISTKYRYDIMA
NCU03076.1	DHQQKIANYAVATPEMVNLAINAALNAKPAWESLPFADRAAVFLKAADLISTKYRYELMA
FG01141.1 	DHATTVASYHTATTADVSAAIDAALAAKPAWEALPFADRAAVFLKAADLISTKYRYDIMA
AN1733.1  	SHG-PVATYSNATAKDVQAAIESALEARKSWASTPFADRASVFLKAADLISTKYRYDVMA
          	.*   :*.*  *:.  *. **: ** *:  * : ** ***::**************::**

MG00189.1 	ATMLGQGKNAWQAEIDSAAELCDFLRFNVQYAEEMYAQQPAHNSPGVWN~RVEYRPLDGF
NCU03076.1	ATMLGQGKNAWQAEIDAAAELCDFLRFNVQYAEQLYAQQPAHNSPGVWN~RVEYRPLEGF
FG01141.1 	ATMLGQGKNAWQAEIDAAAELADFLRFNVHYAEQLYSIQPEHNSPGVWN~RLEYRALEGF
AN1733.1  	LTMHGQGKNAWQAEIDSAAELCDFFRFGVKYAEDLYAQQPVHHAPGVWN2RVEYRPLEGF
          	 ** ************:****.**:**.*:***::*: ** *::***** *:***.*:**

MG00189.1 	VYAVSPFNFTAIAGNLPGAPALLGNVVVWKPSDFAIASNWLLYNILLEAGLPKGVIQFVP
NCU03076.1	VYAVTPFNFTAIAGNLPGVAALLGNVVVWKPSDSAILSNYLLYKVLLEAGLPPDVIQFIP
FG01141.1 	VYAVSPFNFTAIAGNLPGAPALLGNVVVWKPSDFAIASNWLVYQILIEAGLPKDVIQFVP
AN1733.1  	VYAISPFNFTAIGGNLAGAPALMGNVVVWKPSPSAIASNWLVHQILLEAGLPKNVIQFVP
          	***::*******.***.*..**:*********  ** **:*::::*:***** .****:*

MG00189.1 	GDPEEVTRVVLSHRQFAALHYTGSTAIFRKLYGQIGAGVAEGKYASYPRIVGETGGKNFH
NCU03076.1	GDPELVTKEVLAHKDFAALHYTGSTAVFRKLYGQIGAGVAEGRYRSYPRIVGETGGKNFH
FG01141.1 	GNPVDITKVVLEHKEFAALHYTGSTSVFRQLYGTIGQGIAEGRYRGYPRVVGETGGKNFH
AN1733.1  	GEAEEVTKTVLDHPDFAALHFTGSTNVFRNLYGQISTRVAAGKYRSYPRIVGETGGKNFH
          	*:.  :*: ** * :*****:**** :**:*** *.  :* *:* .***:**********

MG00189.1 	LVHHSADLENAVIQTVRGAFEYQGQKCSATSRLYVPKSMWPRFRERLAEEVKKLKIGDPG
NCU03076.1	LVHKSADLRNAVVQTVRGAFEYQGQKCSATSRLYVAKSIWPDFQELLVQETEKLKVGPPH
FG01141.1 	LIDPTAEIDNAVKQTVRGAFEFQGQKCSATSRLYVPKSMWPEFKEKLVAEVEAIKIGNPT
AN1733.1  	LIHKSADIRNAAVQTVRGAFEYQGQKCSATSRVYVASSIADSFLEQVASEAKSLKVGPPS
          	*:. :*:: **. ********:**********:**..*:   * * :. *.: :*:* * 

MG00189.1 	DHGNFIGPVIHEGSFKKLSGVIDEAKNDSKLKLVAGGKYDSSKGYFVHPTIYETTDPAHK
NCU03076.1	DHGNFMGPVIHAASFKKLSTAIDEGKKDPDLQLIAGGEYDSSVGYYVKPTIFVSKTPDHK
FG01141.1 	EHFNFMGPVIHEASFKKLSGAIDEAKSDKDLELVVGGKYDSSKGYYVHPTIYATTNPNHK
AN1733.1  	DFTNFCGPVIHEASFTKLAKVIDEAKNDPELELLAGGSYDSSKGWYIQPTVYRTTNPDHP
          	:. ** ***** .**.**: .***.*.* .*:*:.**.**** *::::**:: :. * * 

MG00189.1 	LLSQELFGPVLVAYAYDDDLHGDPAAAFGKICETVDSTSEYGLTGAVFAVDRAAVRFAED
NCU03076.1	LFSTELFGPVLTVYVYDDYVD----STTS-IYDIIDGATEYGLTGSIFASDRSVIRFAEE
FG01141.1 	FFSTEFFGPILTTYIYDDAAP----NAMANVCKLIETTSDYGLTGAVFAADREASRFAEE
AN1733.1  	LLTRELFGPILVVYAYPDATE----ADFARIAQKIDATGEYGLTGSVFAQDREALAVAND
          	::: *:***:*..* * *          . : . :: : :*****::** ** .  .*::

MG00189.1 	KLRNSAGNFYVNCKSTGAVVGQQPFGGGRASGTNDKAGSMNLLGRFVSTRSLKEEFNATT
NCU03076.1	KLRNSAGNFYINCKSTGAVVGQQPFGGSRASGTNDKAGSINLLSRFVSMRSIKEEFNSTT
FG01141.1 	HLRNAAGNFYVNCKSTGAVVGQQPFGGSRASGTNDKAGSQNLLTRFVNVRAIKEEFVPTT
AN1733.1  	VLRNAAGNFYINCKSTGAVVGQQPFGGARASGTNDKAGSGNLLSRFVSLRSIKEEFVPTY
          	 ***:*****:****************.*********** *** ***. *::**** .* 

MG00189.1 	SVLYPSNQV-
NCU03076.1	TVTYPSNEVE
FG01141.1 	KVTYPSNEV-
AN1733.1  	KVAYPSNEA-
          	.* ****:.
```
